# Application of Wood’s Lamp in Dermatological and Dental Photodiagnostics

**DOI:** 10.3390/s25113253

**Published:** 2025-05-22

**Authors:** Mirosław Kwaśny, Paulina Stachnio, Aneta Bombalska

**Affiliations:** 1 Institute of Optoelectronics, Military University of Technology, Gen. S. Kaliskiego 2, 00-908 Warsaw, Poland; miroslaw.kwasny@wat.edu.pl; 2 Faculty of Mechanical Engineering, Military University of Technology, Gen. S. Kaliskiego 2, 00-908 Warsaw, Poland

**Keywords:** wood’s lamp, fluorescence, enamel, photodiagnosis, dermatology

## Abstract

This article provides an overview of the possibilities of using a modern Wood’s lamp in dermatological diagnostics. In the experimental part, this study examined the possibilities of the light source in photodiagnostics combined with the photodynamic method (PDT) and determined the lower detection limit of the basic photosensitizer in dermatology—protoporphyrin IX (PPIX). The absorption parameters and fluorescence quantum efficiency of PPIX were investigated, and case studies of patients in clinical conditions were presented. A new application may be the use of a Wood’s lamp in dental diagnostics to detect the early stage of caries and to monitor bacterial plaque. Fluorescence area studies were conducted on 21 extracted teeth with different levels of caries. The results showed that changes in enamel demineralization can be detected at a level as low as a 5–10% decrease in fluorescence.

## 1. Introduction

A Wood’s lamp is an inexpensive, easy-to-use, and safe tool in dermatology. It is used to diagnose and monitor a range of fungal and bacterial infections, pigmentary conditions, metabolic disorders, and cutaneous surgery and cosmetics [1]. In addition to dermatology, the lamp is also used in emergency medicine, forensics, ophthalmology, gynecology, and veterinary medicine [2,3,4,5].

The Wood’s lamp was invented in 1903 by Baltimore physicist Robert Wood (1868–1955) [6], and its first documented use in dermatology occurred in 1925 when it was recommended for detecting fungal infections of the hair [7]. This year marks the 100th anniversary of the use of this source in medicine.

A Wood’s lamp was originally a mercury vapor lamp equipped with an optical filter made of barium silicate with the addition of 9% nickel oxide. This filter blocked most of the visible electromagnetic spectrum and allowed ultraviolet light (UVA) to pass through, with a peak emission at a wavelength of 365 nm [8]. A major limitation of the classic Wood’s lamp was its low radiation power density, which required tests to be conducted in heavily darkened rooms.

Modern Wood’s lamps are constructed using a matrix of light-emitting diodes (LEDs) that emit radiation at a wavelength of approximately 365 nm, with significantly higher power densities. As a result, there is no longer a need for a Wood filter, although the name of the lamp has persisted. The primary application of a Wood’s lamp remains in dermatological diagnostics. The review section comprehensively describes the range of diagnostic possibilities using this source.

The experimental part shows that a Wood’s lamp can serve as an effective source of fluorescence excitation for imaging various dermatological changes using the photodiagnostic method (PDD-Photodynamic Diagnosis which complements the photodynamic treatment method. The PDD/PDT method has been dynamically developed over the last few decades in many centers around the world [9,10], including Polish centers [11,12]. The method involves the external introduction of photosensitizers or their precursors into the body, which selectively accumulate in diseased tissues. When these areas are irradiated with light and exposed to internal oxygen, tissue destruction occurs. The PDD method plays an important role in accurately identifying the area to be irradiated, determining the level of accumulated sensitizer over time after its application, and establishing the optimal time to continue tissue irradiation. In dermatology, 5-aminolevulinic acid (ALA) is used in the PDD/PDT method, which is a precursor of the actual photosensitizer—protoporphyrin IX (PPIX). One of the aims of this work was to estimate the lower limit of the PPIX concentration level using a Wood’s lamp.

The results of the PPIX fluorescence analysis in vitro and examples of fluorescence imaging using a Wood’s lamp in clinical conditions are presented. The absorption coefficients of PPIX and its fluorescence quantum yield were determined. One of the technical problems of the PDD/PDT method is the search for cheap and efficient light sources. It was shown that a Wood’s lamp can be an attractive alternative to much more expensive illuminators.

The second new application of a Wood’s lamp may be dental diagnostics to detect early stages of caries and bacterial plaque. The quantitative enamel fluorescence (QLF) method was introduced 20 years ago [13,14] and continues to be developed. [15,16,17]. The most commonly used are expensive enamel fluorescence imaging systems with blue excitation. Using a Wood’s lamp to excite enamel fluorescence, capture images of demineralized areas with a regular mobile phone camera, and analyze them with appropriate software programs can be an attractive alternative to commercial fluorescence systems.

## 2. Review of the Applications of a Wood’s Lamp in Dermatology

### 2.1. Pigmentation Disorders

The main symptoms of pigmentation disorders are hypopigmentation spots, hyperpigmentation spots, leukoderma, and pseudoleukoderma. Hypopigmentation spots are places where there is a lack of melanin, which is caused by the loss of melanocytes themselves. A Wood’s lamp causes autofluorescence of white areas of dermal collagen ranging from light blue to white [18]. Hyperpigmentation manifests itself as darker spots. It often occurs in middle-aged women, particularly on the face and neck, which are the areas most exposed to sunlight, but also to cosmetics and perfumes [19].

Progressive macular hypomelanosis is characterized by discolored spots on the trunk, less frequently occurring on those reaching the limbs or neck. When examined with a Wood’s lamp, it shows red vesicular fluorescence in places where hypopigmented spots occur. Outside these areas, the described phenomenon is not observed [20]. Vitiligo is the loss of melanocytes from the skin and hair, which in turn leads to depigmented spots of various shapes and colors. The two main mechanisms that lead to the loss of melanin are leukoderma, i.e., impaired melanocyte function, and hypopigmentation, i.e., loss of melanocytes themselves [21]. Lesions in a Wood’s lamp are visible as light that is bluish white with a sharp demarcation. Using this diagnostic method, it is also possible to distinguish vitiligo from other changes, such as pityriasis versicolor, anemic nevi, or pityriasis alba [22].

Melasma (chloasma) is an acquired skin discoloration associated with increased melanin production, occurring in areas exposed to the sun. Under the light of a Wood’s lamp, epidermal melasma appears as a sharply demarcated brown or black spot, while cutaneous melasma presents an unaccented, gray-blue discoloration [23].

Epidermal melasma shows increased color contrast when examined under the light of a Wood’s lamp, compared to visible light. Cutaneous melasma, which may have a slightly bluish hue in natural sunlight, does not fluoresce under a Wood’s lamp. Melasma in patients with darker skin (skin types V and VI) is more visible under visible light than under UV light.

### 2.2. Wood’s Lamp in the Diagnosis of Skin Infections

The bacterium *Cutibacterium acnes* (formerly Propionibacterium acnes) naturally occurs on the skin and, when present near the sebaceous glands, can lead to acne vulgaris [24]. *Cutibacterium acnes* is the cause of progressive macular hypomelanosis (PMH). PMH is a hypopigmentation disorder with symptoms similar to pityriasis versicolor and postinflammatory hypopigmentation [25]. To confirm the diagnosis of PMH, a Wood’s lamp is used in the area of the hair-sebaceous follicles, where *Cutibacterium acnes* emits orange-red fluorescence [1]. Due to its presence on the skin, it often causes postoperative infection of eye diseases or during implantation of prostheses and endoprostheses [26,27].

*Pseudomonas aeruginosa* is a multidrug-resistant bacterium. It causes local infections after burns and onycholysis (detachment of the nail plate) or infections of the interdigital spaces [28]. Pseudomonas secretes fluorescein, a phosphor that glows green under a Wood’s lamp. Thus, green fluorescence, in the proper clinical context, indicates infection [29,30,31]. Erythrasma is a chronic infection caused by the bacterium *Corynebacterium minutissimum*. The stratum corneum of the epidermis is attacked, where the bacterium multiplies, leading to keratinization of the epidermis. *Corynebacterium minutissimum* produces the phosphor coproporphyrin III, which produces coral-pink fluorescence. Other conditions are often confused with erythrasma. An example is intertrigo, which also involves changes in the skin folds. It is caused by skin rubbing against skin, leading to a lack of air circulation [32]. Wood’s lamp gives a negative result in the case of intertrigo. Candidiasis has similar risk factors to erythrasma, but here, too, a Wood’s lamp gives a negative result. *Trichomycosis axillaris* is a superficial bacterial infection in which small deposits form on the axillary hair. The disease is usually associated with the bacterium Corynebacterium tenuis [33]. Instead of the coral-red fluorescence seen in erythrasma, *Trichomycosis axillaris* shows pale yellow fluorescence under Wood’s lamp [34].

Fungi that cause mycosis of the skin, hair, and nails are divided into three genera: *Microspora*, *Trichophyton*, and *Eidermophyte*. Of these, fluorescence can be observed when examining *Microsporum* and several species of Trichophyton. The former produces a blue-green fluorescence from the pteridine porphyrin. On the other hand, *T. schoemleinii* from the Trichophyton genus produces blue fluorescence, and *T. verrucosum* from the same genus fluoresces in cattle [1].

Light green fluorescence is seen in infections with *Microsporum audouinii*, *Microsporum canis*, *Microsporum distortionum*, *Microsporum ferrugineum*, and *Microsporum gypseum*.

Tinea capitis is a dermatophyte infection of the hair follicle and the surrounding skin. Characteristic fluorescence is usually seen in broken-off hairs and in the interfollicular part when the hair is pulled out [35].

Tinea capitis, which was previously most often caused by *Microsporum audouinii* and easily diagnosed with a Wood’s lamp, is now predominantly caused by Trichophyton tonsurans, which does not fluoresce [1].

The exception is *Trichophyton schoenleinii*, which causes blue fluorescence. This genus is the causative agent of tinea favosa (Latin: favus), which is a severe form of tinea capitis. Favus is defined as tinea capitis. It can also affect the beard, nails, and body. Tinea capitis of gray patches is visible on Wood’s lamp examination as yellow-green fluorescence due to pteridines present in hairs infected with dermatophytes of the *Microsporum* genus. Pityriasis versicolor (Latin: pityriasis versicolor) is a mild and non-infectious but chronic mycosis caused by Malassezia [36]. Pityriasis versicolor is caused by *Malassezia globosa*, a lipid-dependent fungus. There is epidermal proliferation caused by free fatty acids (FFA), which disrupt the scalp barrier by penetrating the stratum corneum [37]. During a Wood’s lamp examination, yellow-white or copper-orange fluorescence is visible in active classic infection, while blue-white fluorescence is visible in the vesicular form. Diagnosis of tinea versicolor with a Wood’s lamp is challenging, as studies have shown that only one-third of the lesions exhibit fluorescence, primarily due to the washing away of chromophores during bathing before the examination. In addition, cosmetics or sunscreens may also give a false positive result [37].

### 2.3. Other Uses of a Wood’s Lamp in Dermatology

A Wood’s lamp produces a characteristic pattern in certain cutaneous porphyrias, which result from enzymatic defects in heme synthesis and the subsequent accumulation of porphyrins in tissues, blood, urine, and feces [38]. Specifically, porphyrins produced in porphyria cutanea tarda (PCT) fluoresce pink or orange-red in urine and feces under a Wood’s lamp. Additionally, urine, blood, and teeth fluoresce bright coral due to the high levels of uroporphyrin I and coproporphyrin. However, in erythropoietic protoporphyria, the skin, teeth, nails, and urine do not fluoresce, although erythrocytes are also affected [22].

Milia are cysts formed by keratin retained by the dermis. Keratin fluoresces bright yellow under a Wood’s lamp. It is also possible to test for porokeratosis, granuloma annulare, and lichen planus annulare. Porokeratosis is a hereditary disorder that manifests itself as keratinization of the epidermis. It causes blue-brown fluorescence with white edges, and its shape is similar to a rhombus [1]. Wood’s lamp has been used in: assessing scabies, detecting drugs administered systemically (e.g., tetracycline or mepacrine in skin and nails), detecting allergens on the skin in cosmetic allergies, determining the circulation time of fluorescein injection, detecting semen in cases of sexual abuse and treating warts in children [39,40]. It is possible to diagnose photoallergic dermatoses, which are a delayed-type reaction caused by chemical substances resulting from the absorption of radiation, mainly UV [41].

## 3. Materials and Methods

### 3.1. Measuring Apparatus

The fluorescence and emission spectra of a Wood’s lamp were studied using an LS900 fluorimeter (Edinburgh Inst., Livingstone, Scotland). The spectra were measured with a resolution of 2 nm. The excitation source was a Xenon lamp with an emission range of 200–2000 nm. Both excitation and emission parameters can be chosen within this range through the monochromator’s setup. One monochromator is placed in front of the measuring chamber; the other one is directly behind. Such construction allows the operator to regulate the recording of the required wavelength. The absorbance of the solutions was measured using a Lambda 900 spectrophotometer from Perkin-Elmer (Waltham, MA, USA) with a resolution of 1 nm.

**Figure 1:**
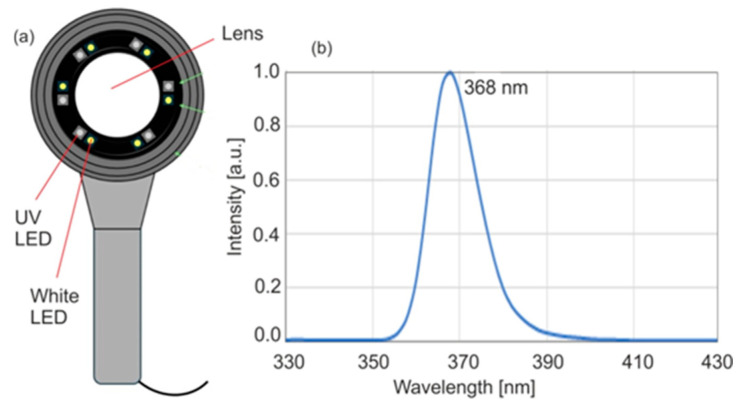
View of Wood’s lamp (**a**) and emission spectrum (**b**).

A Wood’s lamp model KN-9000B (Kernel Medical Equipment Co., Ltd., Xuzhou, China) was used. The view and the measured radiation emission spectrum are shown in Figure 1. The power density measurements were performed using a DET10 A2 Thorlab meter (Newton, NJ, USA).

The lamp consists of six light-emitting diodes (LEDs) emitting radiation at a wavelength of 365–370 nm, centrally located around the circumference, along with six white light LEDs. A lens is placed above the surface of the diodes to double the image magnification. A plastic limiter ensures a constant distance of 5 cm from the source surface to the tested object. The fluorescent image is recorded using a camera in the phone. The device has three lighting intensity modes for UV radiation and white light. The lamp is powered by a battery, which makes the device portable, but a constant power source is necessary for this.

The maximum emission is 368 nm, which is consistent with the manufacturer’s data. The radiation power densities in different irradiation modes are 0.32, 0.82, and 1.2 mW/cm^2^, respectively, which means a value many times greater compared to the classic Wood’s lamp.

### 3.2. Analysis of Fluorescent Images

**Figure 2:**
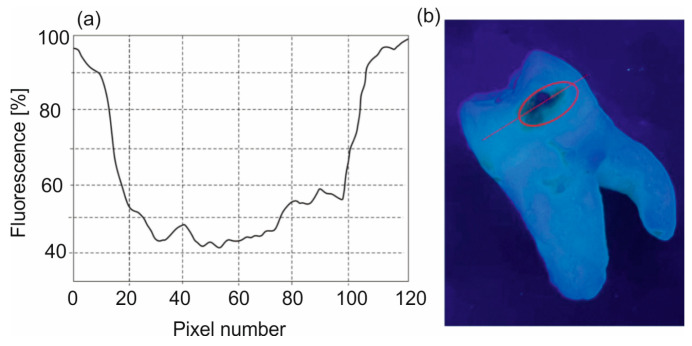
Analysis of the fluorescent image of tooth enamel with caries: (**a**) fluorescence intensity and (**b**) fluorescent image of a tooth with marked caries.

The analysis of the fluorescence level in fluorescent images obtained using a Wood’s lamp was performed using the ImageJ for Windows V 1.54 program. The color image consists of a combination of primary colors—red (R), green (G), and blue (B). The RGB color channels were separated, and a straight line was drawn along the carious lesion to obtain the distribution of fluorescence intensity as a function of pixel position. An example of such a procedure is shown in Figure 2. The blue color channel was used for enamel analysis, and the red color was used for PPIX marking.

The results show the ratio of the minimum and maximum values obtained along the line for each of the enamel photos. The obtained value was subtracted from unity and presented as a percentage. This way, the value of fluorescence reduction in carious areas was obtained. The methodology of analyzing the decrease in the fluorescence level of enamel consists of determining the ratio of the emission intensity of enamel changed by caries in relation to healthy enamel. This ratio does not depend on the intensity of light that excites fluorescence or the geometric conditions of measurements. The fluorescence intensity is analyzed on the image along the line passing through the point with the greatest decrease in fluorescence. Hence, the results are very repeatable. It is similar to the analysis of images from PPIX.

### 3.3. Preparation of PPIX Solutions

A 56.7 mg weight of PPIX from Porphyrin Products was dissolved in 10 cm^3^ of 0.1 M NaOH and adjusted to pH = 7.2 by adding 0.1 N HCl. The solution was diluted in a flask to a volume of 100 cm^3^, resulting in a starting solution with a molar concentration of 1 × 10^−3^ M (mol/dm^3^), which corresponds to a concentration of 0.567 mg/cm^3^. Then, a series of solutions diluted with PPIX in the range of 10^−4^–10^−7^ M were prepared.

### 3.4. Clinical Studies of Patients

Clinical studies were conducted at the dermatological clinic on a group of twenty patients with a lesion, sinilis keratosis (solar keratosis), two with warts, and ten with basal cell carcinoma. The preparation used was AMELUZ (7.8% concentration of 5-aminolevulinic acid) in the form of a gel from Biofrontera, Leverkusen, Germany. A layer of cream was applied to the skin lesion and secured with a bandage to prevent accidental abrasion from the skin. After specific time intervals following application, the diseased skin was illuminated with a Wood’s lamp, and the fluorescence image of biosynthesized PPIX was captured using a mobile phone camera (Samsung, Suwon, Republic of Korea).

### 3.5. Teeth Material

In vitro studies on the use of a Wood’s lamp in dentistry were conducted on 21 human teeth extracted for various dental reasons. Teeth with an intact crown and without clinical caries, but with carious spots, were selected for the study. A total of 31 areas of enamel affected by caries were analyzed.

### 3.6. Criteria for Determining Fluorescence Reduction or Detection Limits

According to the literature data, the concentration of PPIX in the body after ALA application is in the range of 5 × 10^−5^–1 × 10^−6^ M. We therefore adopted the criterion that the detection limit of the lamp should be one order of magnitude lower. Based on the literature [14], it is assumed that in the case of early caries, a 5–10% enamel loss allows for enamel remineralization using an appropriate paste, which prevents the need for drilling. One of the aims of the work was to investigate what minimum level of fluorescence intensity decrease is possible for analysis using a Wood’s lamp. A color CCD camera of a mobile phone was used to record fluorescent images. Such cameras, compared to other fluorescence imaging systems (e.g., MCP, ICCD, streak cameras, etc.), have low sensitivity. Hence, it was necessary to check whether this simple experimental system is sufficiently sensitive to such a low decrease in enamel fluorescence.

### 3.7. Research Framework

The research in the article was divided into a part concerning fluorescence photodiagnostics of enamel. They determined the excitation characteristics of healthy enamel, the effect of the excitation wavelength on the position of the fluorescence spectrum, changes in the emission intensity of enamel with different degrees of caries when excited with 368 nm radiation (corresponding to a Wood’s lamp), measurements of fluorescence images of extracted teeth, analysis of the decrease in fluorescence of carious enamel, and statistics of the obtained results. In the part concerning PDD/PDT photodiagnostics, a series of PPIX solutions in a wide range of concentrations was prepared, the detection limit of PPIX using a Wood’s lamp was determined, the relative quantum efficiency of fluorescence was examined using the method and examples of fluorescence imaging were presented in a group of patients with selected dermatological changes.

## 4. Results

### 4.1. Enamel Fluorescence Studies

**Figure 3:**
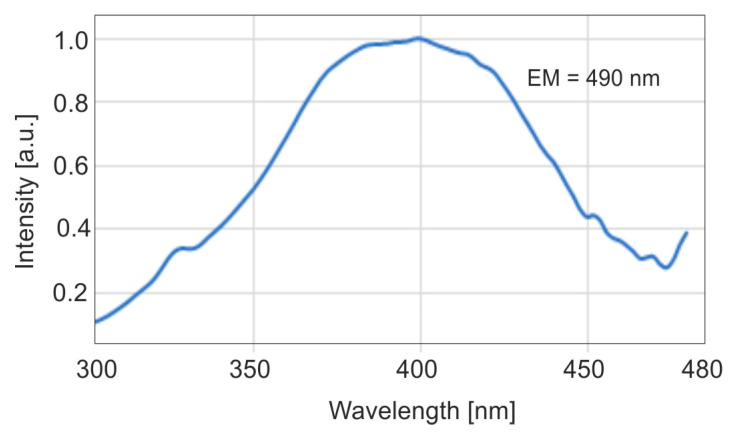
Enamel fluorescence excitation spectrum (EM = 490 nm).

The emission characteristics of enamel, presented as emission-excitation (EM-EX) maps and fluorescence spectra, were analyzed after excitation with lasers at wavelengths of 407, 442, and 633 nm using a fiber optic analyzer [42]. At each excitation wavelength, the fluorescence intensity from carious lesions is significantly lower than that of healthy enamel. The interesting fluorescence range of the present study concerns the UV-VIS range. Tooth enamel exhibits strong fluorescence when excited with radiation in the wavelength range of 300–480 nm. Figure 3 shows the excitation spectrum of enamel at 490 nm emission. The maximum excitation wavelength is approximately 400 nm.

**Figure 4:**
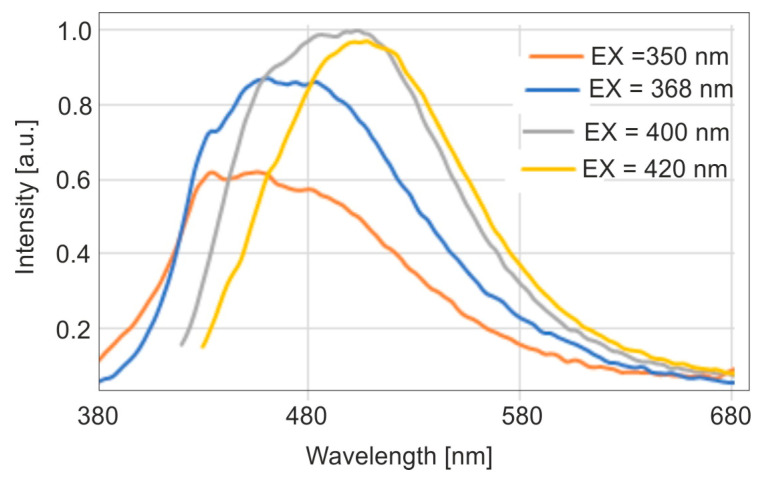
Emission spectrum of enamel for different excitation wavelengths.

For healthy enamel, fluorescence measurements were performed with excitation of radiation at wavelengths of 350, 368, 400, and 420 nm using a fluorimeter. The wavelength of 368 nm corresponds to the emission of a Wood’s lamp. The fluorescence maximum was achieved with excitation at a wavelength of 400 nm. However, with excitation at 368 nm, the fluorescence intensity reached 87% of the maximum level. This indicates a very good spectral match of a Wood’s lamp to enamel fluorescence studies. The maxima of the emission bands shift towards longer wavelengths with the increase of the wavelength of the excitation radiation. The measurement results are shown in Figure 4.

**Figure 5:**
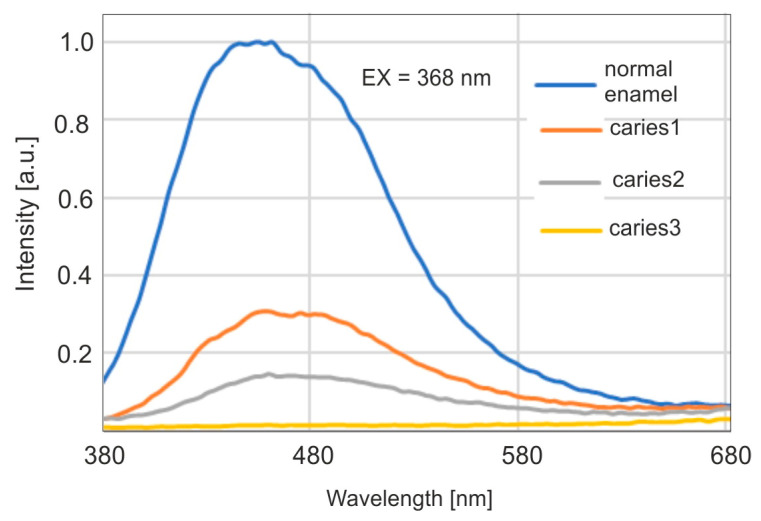
Emission of fluorescence of enamel at various stages of caries advancement with excitation at 368 nm.

Fluorescence spectra were then studied for carious enamel with excitation of radiation at a wavelength of 368, corresponding to the emission of a Wood’s lamp. The results are presented in Figure 5.

A significant decrease in fluorescence intensity is observed for subsequent, deepening stages of caries. At the same time, a shift of the maximum towards longer wavelengths is visible. The maximum for carious enamel occurs around the wavelength of 460 nm, which is visible as blue. It is therefore possible to use a Wood’s lamp in a dental office as a dentist’s tool to detect caries at an early stage, due to the visibility of the emitted fluorescence.

**Figure 6:**
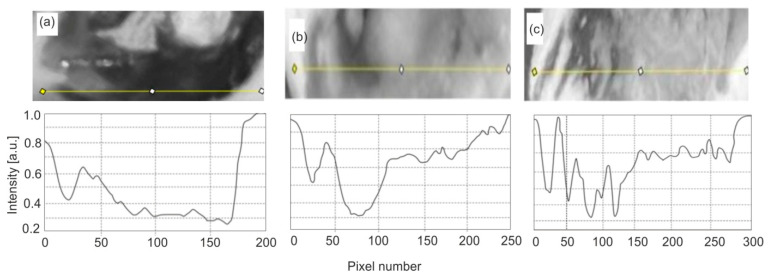
Decreases in fluorescence of enamel with caries: (**a**) very high decrease, (**b**,**c**) medium decrease.

Figure 6 presents examples of distributions of fluorescence image intensity along the carious lesion. Fluorescence values were calculated from the ratios of the intensity in individual pixels between carious and healthy enamel.

The conducted studies show that the area of caries is characterized by large inhomogeneities in changes. The calculation of the fluorescence decrease can be performed in two ways: as an average value over the entire pixel range by measuring the ratios of the fluorescence area of healthy (100%) and carious enamel. The more important parameter is the value of the maximum fluorescence decrease. The area of the defect is less important and does not affect the decision on further treatment. In relation to the calculated average, this method of calculation gives higher values of the fluorescence decrease. This value indicates the maximum thickness of the enamel caries layer.

**Figure 7:**
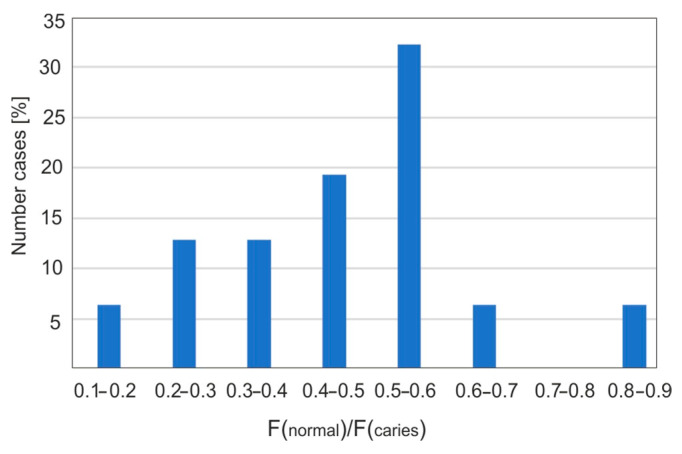
Fluorescence reduction ranges and their percentage share.

Figure 7 presents the statistical percentage of carious teeth as a function of intervals with a specific percentage of fluorescence decrease. The largest fluorescence decrease values are in the range of 50–60%. This may be due to the fact that the samples used were extracted teeth, most of which exhibited caries of considerable size and an advanced stage. Only some teeth showed early signs of caries in the form of white spots, known as “white spot lesions”. In fluorescence images, a noticeable decrease in intensity, around 10%, was observed. This demonstrates the high accuracy and effectiveness of using a Wood’s lamp in the dental diagnosis of early-stage caries.

**Figure 8:**
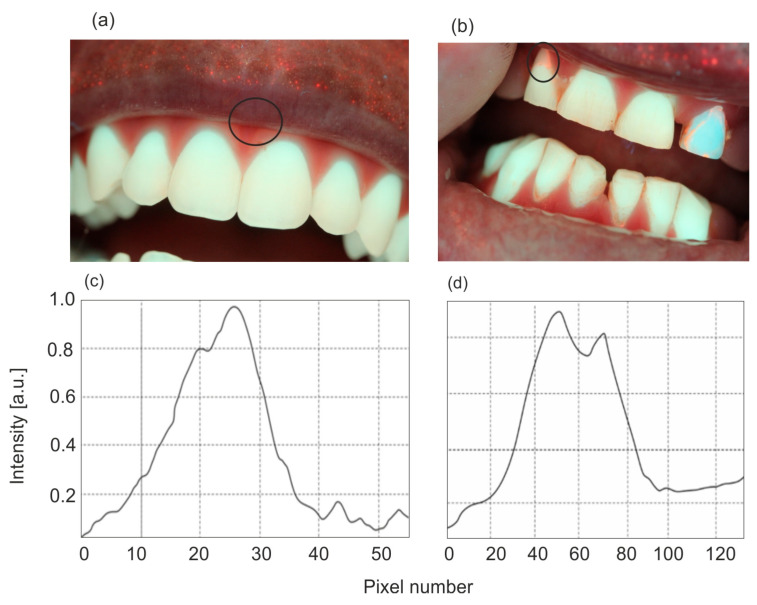
Red fluorescence of bacterial plaque (**a**,**b**) and intensity of fluorescence of porphyrins (**c**,**d**).

An important advantage of fluorescent enamel analysis is the possibility of early detection of dental calculus. Bacteria in the oral cavity produce porphyrin derivatives, which, when excited with violet light or UV radiation, produce strong, red fluorescence. Figure 8 shows sample images of enamel with dental calculus.

### 4.2. Studies of Absorption and Fluorescence Properties of PPIX

**Figure 9:**
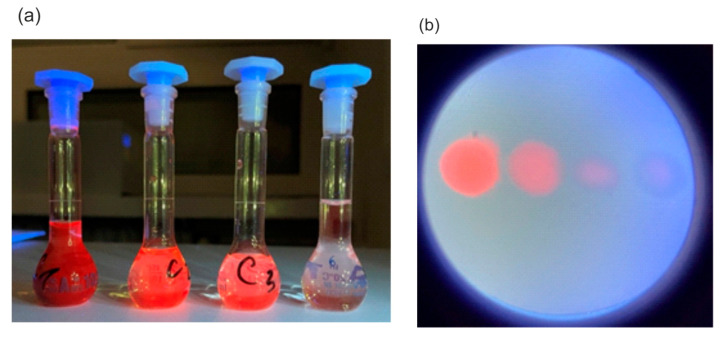
Excitation of fluorescence of PPIX with Wood’s lamp (**a**) in solution and (**b**) on the filter bible.

The final goal of this part was to estimate the lower limit of detection of biosynthesized PPIX in the body after external application of ALA. Figure 9 presents fluorescence images of prepared PPIX solutions in flasks and on filter papers. A Wood’s lamp was positioned 5 cm from the samples, operating at its highest power of 1.2 mW/cm^2^. The estimated detection limit of PPIX concentration using the visual method is 1 × 10^−7^ M (0.053 µg/cm^3^), which is two orders of magnitude lower than the typical PPIX concentration observed four hours after application.

**Figure 10:**
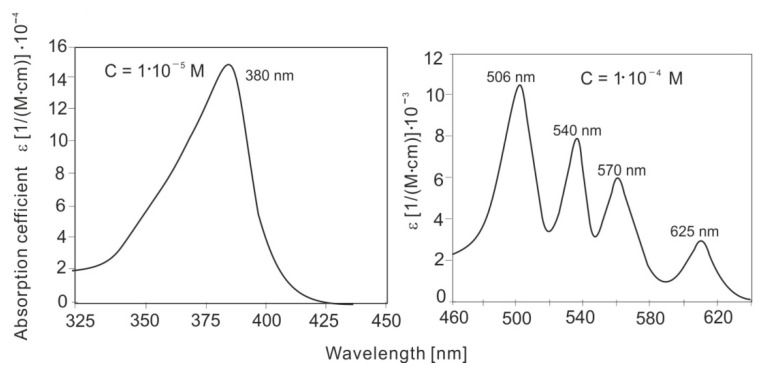
Molar absorption coefficients of PPIX.

Based on the absorbance measurements of PPIX solutions, its molar absorption coefficients in the UV-VIS range were calculated. The compound is characterized by a strong Soret absorption band with a maximum at a wavelength of 380–385 nm and four absorption bands, one order of magnitude weaker, at wavelengths of 506, 540, 570, and 625 nm. The calculated molar absorption coefficients of PPIX are shown in Figure 10.

**Figure 11:**
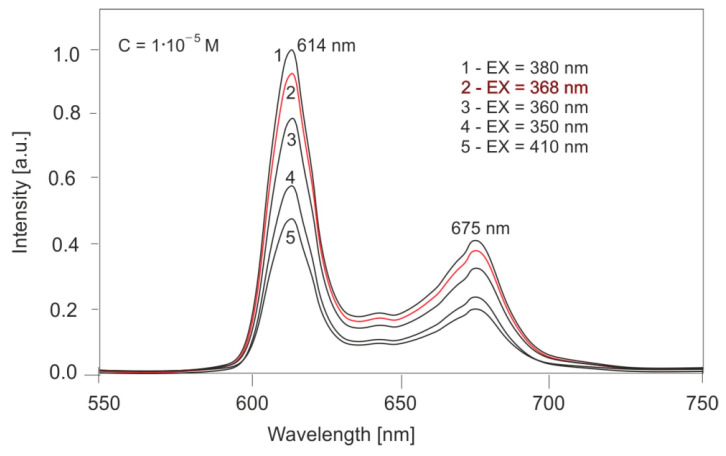
Effect of excitation wavelength on fluorescence intensity.

The next step was to investigate the emission properties of PPIX. Figure 11 shows the effect of the excitation wavelength on the fluorescence spectra of PPIX solutions. For a concentration of 10^−5^ M, excitation wavelengths of 350, 360, 368, 380, and 410 nm were used. The compound exhibits two emission bands at 614 and 675 nm. The position of these bands does not change with the excitation wavelength; only the band intensities change proportionally to the absorbance value. For excitation with a wavelength of 368 nm, corresponding to a Wood’s lamp, the fluorescence intensity is 94% of the intensity for excitation with radiation at a wavelength of 380 nm (the maximum excitation value).

The effect of PPIX concentration on PPIX fluorescence spectra was also determined. The concentration values of the diluted solutions were as follows: 1 × 10^−5^, 8 × 10^−6^, 6 × 10^−6^, 4 × 10^−6^, and 2 × 10^−6^ M.

**Figure 12:**
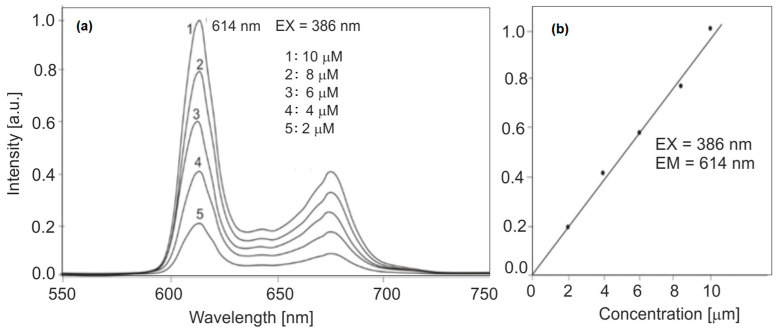
Effect of PPIX concentration on fluorescence intensity (**a**), and standard curve of the effect of PPIX concentration on fluorescence intensity (**b**).

The test results are presented in Figure 12. The change in concentration does not change the fluorescence wavelength, and the fluorescence intensity is linear in relation to the concentration.

The quantum yield of PPIX fluorescence was determined by the relative method based on the quantum yield of the standard—quinine sulfate (QSO4).

Fluorescence quantum yield calculation method:

- sample fluorescence intensity I_s_

I_s_ = I_0_∙k∙A_s_∙ϕ_s_ (1)

where I_0_—incident radiation intensity, k—proportionality factor, A_s_—sample absorbance at 368 nm, and ϕ_s_—sample fluorescence quantum yield,

- standard fluorescence intensity I_st_

I_st_ = I_0_∙k∙A_st_∙ϕ_st_ (2)

where I_0_—incident radiation intensity, k—proportionality factor, A_st_—standard absorbance at 368 nm, and ϕ_st_—standard fluorescence quantum yield.

I_s_/I_st_ = (I_0_⋅k⋅Φ_s_⋅A_s_)/(I_0_⋅k⋅Φ_st_⋅A_st_) = (Φ_s_⋅A_s_)/(Φ_st_⋅A_st_) (3)

Φ_s_ = Φ_st_∙(∫I_s_)/(∫I_st_)∙A_st_/A_s_ (4)

∫_s—denotes the area under the fluorescence curve.

**Figure 13:**
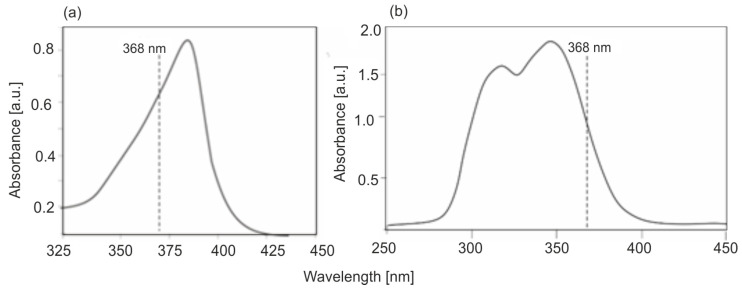
Absorption spectra of PPIX (**a**) and quinine sulfate (**b**).

Figure 13 presents the normalized absorption spectra of PPIX solutions with a concentration of 1 × 10^−5^ M and quinine sulfate with a concentration of 1 × 10^−5^ M. The selection of a standard solution for the tested one is often not easy due to the lack of a common excitation wavelength. In this case, it can be seen that the excitation bands of both PPIX and quinine sulfate enable obtaining strong fluorescence.

Calculation of fluorescence quantum yield:

Φ_st_ = 0.55, A_st_ = 0.12, A_PPIX_ = 0.31, ∫ I_st_ = 3.01 × 10^6^, ∫ I_PPIX_ = 9.85 × 10^5^

The quantum yield of PPIX is low compared to typical organic fluorescent compounds; however, its absorption coefficient is very high, leading to strong fluorescence intensity.

### 4.3. Clinical Studies

**Figure 14:**
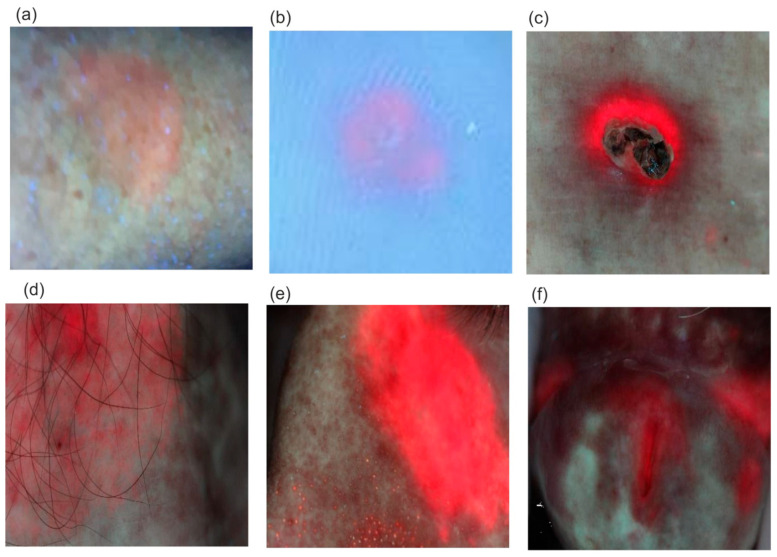
Fluorescent images of diseased areas in patients: (**a**) sililis keratosis, application time—30 min, (**b**) viral warts of the feet, application time—10 h, (**c**) basal cell carcinoma on the back 4 h after application of the preparation (**d**,**e**), sinilis keratosis of the chest 4 h after application of the preparation, and (**f**) sinilis keratosis of the head 4 h after application of the preparation.

Examples of fluorescent images of diseased areas after ALA application and PPIX biosynthesis are shown in Figure 14.

The fluorescence intensity of PPIX increases over time after the preparation is applied. The typical timeframe for reaching the maximum PPIX level, when photodynamic irradiation begins for selective tissue destruction, is 2–4 h. However, PPIX fluorescence can often be observed as early as 30 min after application (Figure 14a). An interesting, previously unpublished case involves viral warts on the feet (Figure 14b), where PPIX secretion requires a significantly longer application time of about 10–12 h. Although the amount of synthesized PPIX is small, it is sufficient to destroy the lesion. Classic applications of the PDD/PDT method include the treatment of basal cell carcinoma and actinic keratosis, with cure rates ranging from 70% to 100%.

## 5. Discussion

A Wood’s lamp tested in this study is designed with concentrically arranged light-emitting diodes (LEDs), emitting radiation with a measured wavelength peaking at 368 nm. A Wood’s lamp, equipped with a concentrically arranged LED matrix emitting at a peak wavelength of 368 nm, demonstrates significantly higher radiation power densities of 0.32 and 0.82 mW/cm^2^ compared to the classic mercury-based Wood’s lamp (*p* < 0.1 mW/cm^2^). This enhanced power allows fluorescence measurements in slightly darkened rooms, eliminating the need for a completely dark environment, which greatly facilitates clinical work. The LED matrix ensures uniform illumination of the examined surface and allows for consistent measurements from a fixed distance. Additionally, it provides a stable platform for capturing fluorescent images using a mobile phone. The lamp’s spectral compatibility with the excitation wavelength of protoporphyrin IX (PPIX) and enamel is notable, with fluorescence intensity reaching 79% of the maximum at 380 nm for PPIX and 87% of the maximum at 400 nm for enamel.

The absorption and fluorescence measurements of PPIX—the initial photosensitizer in the photodynamic treatment (PDT) and photodiagnostics (PDD) methods—were performed, and the spectral parameters were analyzed in terms of the use of a Wood’s lamp for the possibility of detecting PPIX in the body. Based on the absorption measurements of PPIX solutions, its absorption coefficients were calculated. The absorption spectrum of PPIX consists of the strongest Soret band with a maximum at a wavelength of 380 nm and an absorption coefficient of ℇ = 1.5 × 10^5^ dm^3^ mol^−1^ cm^−1^. In the 500–630 nm range, the absorption spectrum contains four weaker bands with wavelengths of 506, 540, 570, and 630 nm and absorption coefficients smaller by an order of magnitude: 1.1 × 10^4^, 8.3 × 10^3^, 6.0 × 10^3^, and 4.3 × 10^3^ dm^3^ mol^−1^ cm^−1^, respectively. For a wavelength of 368 nm, associated with the emission of a Wood’s lamp, the absorption coefficient is 1.1 × 10^5^ dm^3^ mol^−1^ cm^−1^, which is 73% of the maximum absorption coefficient value in the spectrum. A Wood’s lamp is therefore very well spectrally adapted to PPIX analysis.

Based on the measurements of PPIX absorption and fluorescence spectra and the standard, quinine sulfate, the quantum yield of PPIX fluorescence was determined. The quantum yield of PPIX below 0.1 (0.07) indicates that it is a compound with medium fluorescence among organic compounds. However, a very high value of the absorption coefficient determines the high sensitivity of detection by the fluorescence method. The detection limit of PPIX using a Wood’s lamp with visual observation of fluorescence was determined at the level of 1 × 10^−7^ mol/dm^3^ (0.053 µg/cm^3^). It is known from the literature [43] that the concentration of synthesized PPIX in the body under the influence of externally introduced 5-aminolevulinic acid is 200–300 times higher after 4 h of application, and at least 50 times higher after 0.5 h of application. Therefore, a Wood’s lamp is a very effective source of fluorescence excitation in the PDT/PDD method in dermatological diseases.

Carious changes cause a decrease in fluorescence intensity. For 31 carious areas, the most common decrease in fluorescence was 50–60% compared to healthy enamel, which constitutes 32% of all results. The highest fluorescence decrease was obtained at 88%, and the lowest at 10%. However, it is possible to detect caries at a much lower level, even below 5%. This is of great clinical importance because, in the case of early caries, it is possible to remineralize the enamel with effective toothpastes, eliminating the need for drilling.

One of the key advantages of using a Wood’s lamp in dental diagnostics is the early detection of dental calculus, which appears as a bright red fluorescence due to the porphyrins secreted by bacteria present in the calculus. Most dental calculus is visible in the interdental spaces. For the three examples of dental calculus tests, an increase in red fluorescence in the interdental spaces of 50% to 80% was obtained, compared to enamel areas without visible fluorescence. Control and removal of dental calculus is a standard procedure due to the possibility of bacteria entering the treated areas, and is mandatory in the case of root canal treatment.

The conducted studies show that the presented Wood’s lamp can be an alternative solution to expensive and complicated fluorescence imaging systems using a regular mobile phone camera.

## 6. Conclusions

The article examines the emission parameters of a modern Wood’s lamp, confirming its high usefulness in everyday dental and dermatological practice. Several times higher UVA radiation power density compared to a classic lamp allows for the examination of many dermatological conditions described in Chapter No. 2 in conditions of moderate office lighting, without the need to completely darken it. One of the aims of the work was to examine the possibility of using the source in the rapidly developing PDD/PDT method for the analysis of PPIX levels. Doctors performing therapeutic irradiation very rarely perform initial diagnostics and usually use the generally accepted, standard time after ALA application of about 3–4 h. In very many cases, however, PDT procedures must be repeated many times, because the accumulation of PPIX is too small. Control of the PPIX level is necessary to perform a PDT procedure. In many dermatological changes (e.g., viral warts), this time is extended to several hours. An important practical application of a Wood’s lamp may be the diagnosis of enamel and the detection of early stages of caries. Detecting advanced caries is easy using a visual method, but the beginnings of early caries are very difficult to observe. These changes appear on healthy enamel as “white spots,” and the contrast is very small. Using the fluorescence method reverses the contrast, and dark areas appear on the white enamel background, which are easy to observe. Even without analyzing the intensity of the resulting fluorescent image, the degree of enamel loss can be easily assessed. In addition, Wood’s lamp causes strong, red fluorescence of porphyrins accumulated in bacterial plaque. Before each dental procedure, the plaque should be removed without fail to prevent infection. The lamp allows for a precise test of bacterial purity.

## Data Availability

The data presented in this study are available on request from the corresponding author.

## Abbreviations

The following abbreviations are used in this manuscript:

PDT	Photodynamic Therapy
PDD	Photodynamic Diagnosis
PPIX	Protoporphyrin IX
LED	Light-emitting diodes
